# The causal relationship between gut microbiota and constipation: a two-sample Mendelian randomization study

**DOI:** 10.1186/s12876-024-03306-8

**Published:** 2024-08-19

**Authors:** Nan He, Kai Sheng, Guangzhao Li, Shenghuan Zhang

**Affiliations:** 1 College of Medical Technology, Chengdu University of Traditional Chinese Medicine, Chengdu, 610075 Sichuan PR China; 2 Shriners Hospital for Children, Montreal, QC Canada; 3 Sichuan Key Laboratory of Medical Molecular Testing, Chengdu, 610075 Sichuan PR China

**Keywords:** Constipation, Gut microbiota, Mendelian randomization, Causal relationship, GWAS

## Abstract

**Background:**

Constipation is one of the most common gastrointestinal disorders afflicting the population, with recent observational studies implicating dysfunction of the gut microbiota in constipation. Despite observational studies indicating a relationship, a clear causality remains unclear. This study aims to use two-sample Mendelian randomization (MR) to establish a clearer causal relationship between the two.

**Methods:**

A two-sample Mendelian randomization (MR) study was performed using the gut microbiota summary Genome-Wide Association Studies (GWAS) statistics from MiBioGen consortium (*n* = 13,266) and constipation GWAS summary statistics from the IEU OpenGWAS database. The causality between gut microbiota and constipation is primarily analyzed using the inverse-variance weighted (IVW) method and reinforced by an additional four methods, including MR-Egger, Weighted Median, Simple Mode, and Weighted Mode. Finally, funnel plot, heterogeneity test, horizontal pleiotropy test, and leave-one-out test were used to evaluate the reliability of MR results.

**Results:**

IVW estimates suggested that the bacterial species *Anaerotruncus*, *Butyricimonas*, and *Hungatella* were causally associated with constipation. The odds ratio (OR) values of *Anaerotruncus*, *Butyricimonas*, and *Hungatella* were 1.08 (95% CI = 1.02–1.13; *P* = 0.007), 1.07 (95% CI = 1.01–1.13; *P* = 0.015), 1.03 (95% CI = 1.00-1.06; *P* = 0.037) respectively. Meanwhile, *Ruminiclostridium* 9 and *Intestinibacter* have been shown to be associated with a reduced risk of constipation. The OR of *Ruminiclostridium* 9 = 0.75(95% CI = 0.73–0.78, *P* < 0.001 and *Intestinibacter* of OR = 0.89 (95% CI = 0.86–0.93, *P* < 0.001). Furthermore, validation by funnel plot, heterogeneity test, and horizontal pleiotropy test showed that MR results were reliable.

**Conclusion:**

This is the first Mendelian randomization study to explore the causalities between specific gut microbiota taxa and constipation, and as such may be useful in providing insights into the unclear pathology of constipation which can in turn aid in the search for prevention and treatment.

**Supplementary Information:**

The online version contains supplementary material available at 10.1186/s12876-024-03306-8.

## Introduction

Constipation, one of the common gastrointestinal disorders, manifests as hard stool and extended bowl movement cycles, and affects about 2–35% of the population spanning all ages [1, 2]. Severe constipation can lead to significant complications such as rectal bleeding, nausea, vomiting, weight loss, bowel obstruction, fecal impaction, hemorrhoids, anal fissures, and rectal prolapse. These physical ailments can deeply compromise an individual’s quality of life and often contribute to mental stress [3]. Recent epidemiological studies have shown that constipation is independently associated with other adverse clinical outcomes, such as end-stage renal disease (ESRD), cardiovascular (CV) disease, and mortality, possibly mediated by alterations in gut microbiota and an increased production of fecal metabolites [4, 5]. As a multifactorial condition, constipation’s etiology and pathomechanism remain largely obscure, comprising diverse types with unique underlying causes [6]. Current pharmacotherapies such as lactulose, osmotic laxatives, stimulant laxatives, and intestinal secretagogues, often fall short due to adverse reactions and lack of efficacy [7, 8]. The limited understanding of constipation’s underlying mechanisms, coupled with a shortage of effective treatments, underscores the importance of accurately identifying the root causes of constipation. This is essential for developing more effective and personalized treatment strategies to tackle this complex condition.

The gut microbiota plays a pivotal role in body metabolism, immune regulation, and maintaining the intestinal mucosal barrier’s integrity [9]. Due to the clear importance of the gut microbiota in maintaining homeostasis, its dysbiosis is increasingly recognized as a key factor in the development and progression of constipation [10]. The initial understanding of the link between constipation and gut microbes emerged from traditional culture-based microbiological approaches. Khalif et al. [11] identified lower occurrences of Bifidobacterium and Lactobacillus in constipated individuals compared to control groups, along with increases in *Enterobacteriaceae* (for example, *Escherichia coli*), *Staphylococcus aureus*, and fungi. Over the past decade, advancements in intestinal microbial deep sequencing have doubled the coverage of microbiota, thereby offering a more precise view of the structural and functional alterations in gut microbes [12–15]. Despite supporting some earlier culture-based findings, has also brought contradictory results in numerous studies [16, 17]. For example, Kim et al. [18] found decreased levels of Bifidobacteria and Bacteroides in the feces of constipated adults, whereas Tian et al. reported the opposite [19]. Ohkusa et al. identified a reduced number of beneficial bacteria, such as Lactobacillus and Bifidobacterium in constipation patients [17]. In contrast, Du et al. noted that these beneficial bacteria were more common in Parkinson’s patients with constipation [20]. These studies, mostly utilized case-control designs, faced difficulties in confirming the precise timing of exposure and outcome. Additionally, the relationship between gut microbiota and constipation in observational studies is prone to interference by confounding elements such as age, environmental factors, diet, and lifestyle [21]. These challenges significantly hinder the ability of observational studies to establish a causal connection between gut microbiota and constipation.

Mendelian randomization (MR) offers a novel strategy for delving into the causal connection between gut microbiota and constipation. MR is a statistical method gaining wider usage, employing single nucleotide polymorphisms (SNPs) as instrumental variables (IVs) to construct proxies for exposure, enabling the estimation of a causal link between the exposure and disease outcomes [22]. Unlike observational studies, MR leverages the randomness in allelic inheritance, mirrored by the random genotype allocation from parents to offspring, to emulate randomized controlled studies, thereby reducing the influence of confounding factors and reverse causality in establishing valid causal sequences [23]. Several studies employing MR have identified direct causal links between gut microbiota and cardiovascular disease [24], Parkinson’s disease [25], cholelithiasis [26] and preeclampsia-eclampsia [27]. However, the investigation of gut microbiota and constipation through MR has not yet been conducted. In this study, we perform the first two-sample MR analysis of GWAS summary statistics from the MiBioGen and IEU Open Genome-Wide Association Studies (IEU OpenGWAS) consortiums, revealing the causal impact of gut microbiota on constipation.

## Materials and methods

### Study design

**Fig. 1:**
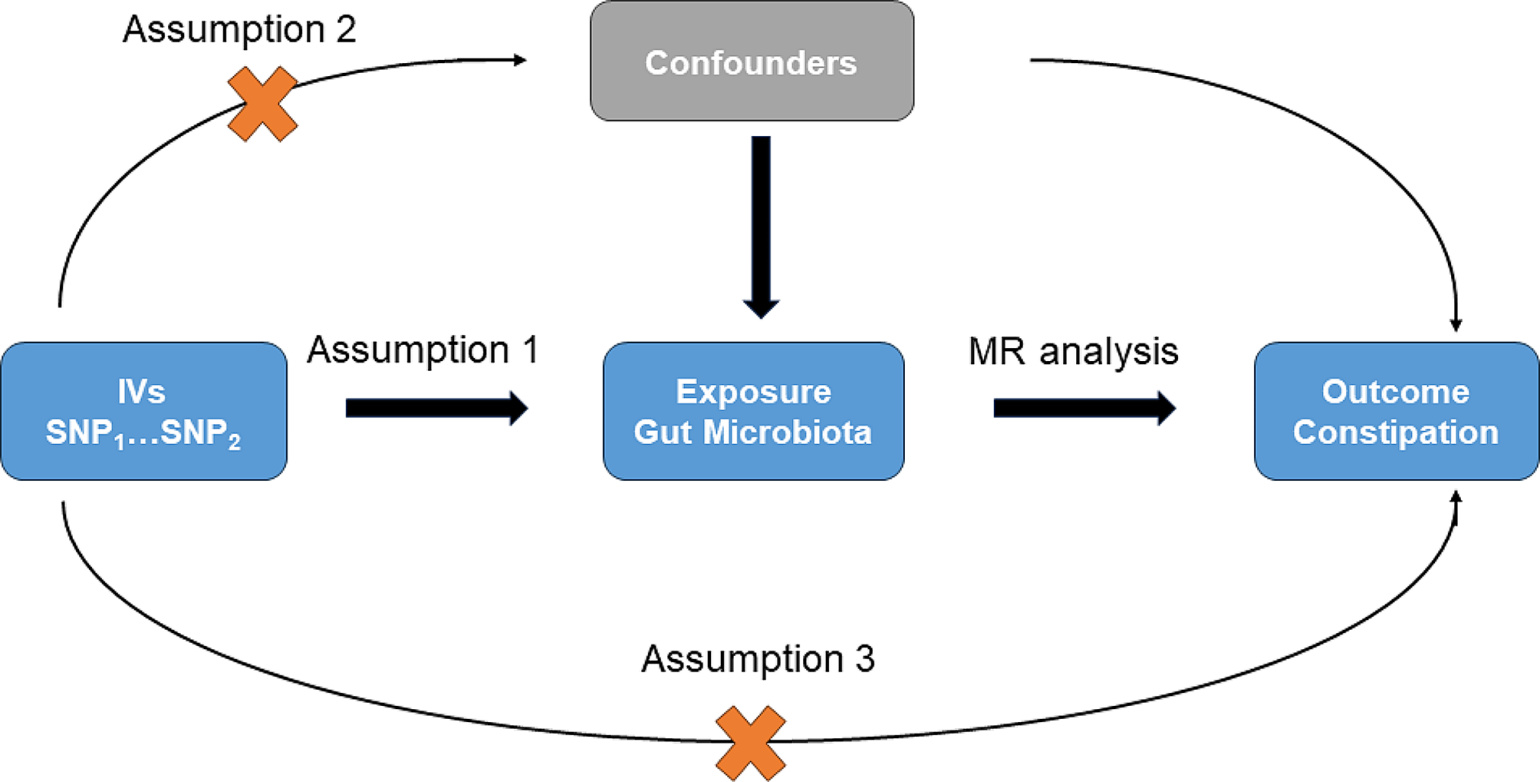
An overview of the study design

We designated constipation as the outcome and considered the exposure to be the gut microbiota for a two-sample MR analysis. To obtain reliable results of causal effects of exposure on the outcome, two-sample MR analysis should satisfy the following three assumptions: first, IVs should be significantly associated with gut microbiota; second, IVs were not associated with any other confounding factors other than gut microbiota; third, IVs only influenced constipation through gut microbiota. The design of this study is outlined in Fig. 1.

### Data source

IEU Open Genome-Wide Association Studies (IEU OpenGWAS) summary (https://gwas.mrcieu.ac.uk/) was used to obtain the summary-level data of constipation, and constipation was used as the outcome. Through search “constipation” in the IEU OpenGWAS database, finn-b-K11_CONSTIPATION (sample: 218,792; the number of single nucleotide polymorphisms (nSNP): 16,380,466) was enrolled in this study. In addition, summary data of gut microbiota including *Ruminiclostridium* 9 (genus.Ruminiclostridium 9.id.11,357; sample: 16,725; nSNP: 978), *Intestinibacter* (genus.Intestinibacter.id.11,345; sample: 12,303; nSNP: 614), *Anaerotruncus* (genus.Anaerotruncus.id.2054; sample: 16,566; nSNP: 518), *Butyricimonas* (genus.Butyricimonas.id.945; sample: 10,737; nSNP: 644) and *Hungatella* (genus.Hungatella.id.11,306; sample: 4209; nSNP: 383), was sourced from MiBioGen (https://mibiogen.gcc.rug.nl/menu/main/home), which contained the largest study in the human microbiome, and were used as the exposure factors.

### Data analysis

To determine a reliable IV, we screened the data extracted from MiBioGen MR using genome-wide significant variants as IVs. The purpose of MR analysis was based on three assumptions: first, IVs should be highly related to the exposure factor only; second, IVs were not associated with any other factors that might be related to both exposure and outcome; third, IVs only influenced the outcome through exposure. To satisfy these three assumptions, SNPs with *P* value < 1 × 10^− 5^ and strong correlation to exposure were selected, and the SNPs with linkage disequilibrium (r^2^ < 0.001, within a 10,000 kb window) were removed by using ‘extract outcome data’ function of ‘TwoSample MR’.

### Data analysis

Based on univariable MR analysis, the causality of gut microbiota on constipation was evaluated. MR analysis was performed through R package ‘TwoSample MR’ (version 0.5.6) [28]. Inverse-variance weighted (IVW) method was performed to estimate the causality of gut microbiota and constipation. Moreover, other four methods (MR-egger, Weighted median, Simple mode and Weighted mode) of MR were used to verify the result of MR.

### Estimation of causal effects between gut microbiota and constipation

Finally, funnel plot, heterogeneity test, horizontal pleiotropy test, and leave-one-out test were used to evaluate whether the MR analysis satisfied these three assumptions through ‘mr heterogeneity’, ‘Horizontal pleiotropy’ and ‘mr leaveoneout’ function of ‘TwoSample MR’.

## Results

### *Anaerotruncus*, *Butyricimonas* and *Hungatella* were associated with constipation

**Fig. 2:**
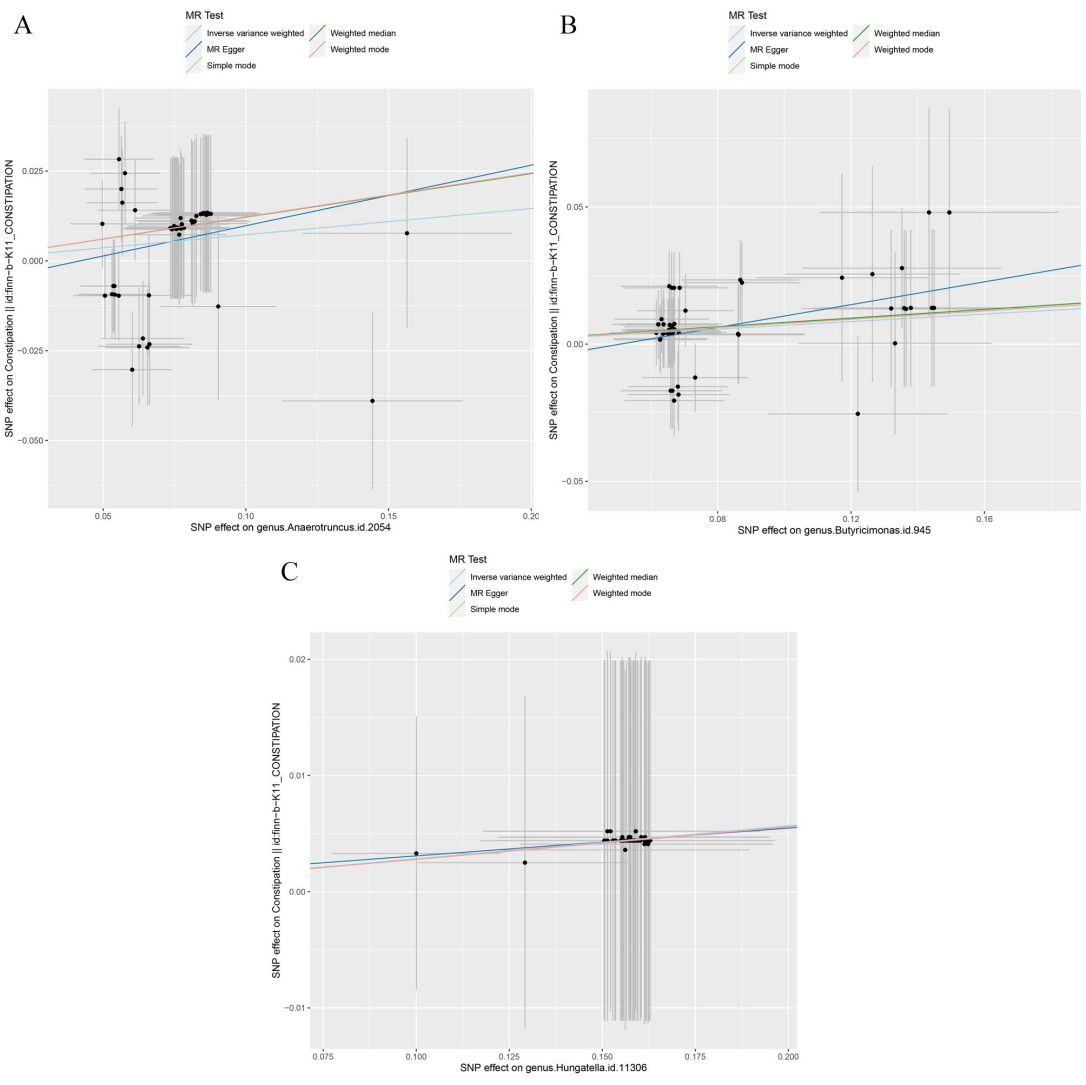
Scatter plots for the causal association between gut microbiota and constipation (**A**) *Anaerotruncus*, (**B**) *Butyricimonas and* (**C**) *Hungatella*

We applied five methods for univariable MR analysis, including Inverse Variance Weighted (IVW), MR Egger, Weighted Median, Simple Mode, and Weighted Mode. Results from all methods showed consensus that *Anaerotruncus*, *Butyricimonas*, and *Hungatella* were causally related to constipation, with β > 0 and OR > 1, as indicated in (Table 1). IVW is the most widely accepted method among these, so we will focus primarily on its results. The odds ratios (ORs) indicated by IVW are as follows: *Anaerotruncus* (OR = 1.08; β = 0.07; 95% CI 1.02–1.13; *P* = 0.007), *Butyricimonas* (OR = 1.07; β = 0.07; 95% CI 1.01–1.13; *P* = 0.015), and *Hungatella* (OR = 1.03; β = 0.03; 95% CI 1.00-1.06; *P* = 0.037) positioning them as risk factors for constipation. The other four methods showed slightly higher ORs and β but wider 95% CI. These results were confirmed by scatter plots (Fig. 2A-C, slopes > 0), which also suggested that no other factors influenced the outcome, as the intercepts for all five MR methods were close to zero.

**Table 1:** Mendelian randomization results of risk factors of constipation

Outcome	Exposure	IVs	Method	β	se	pval	OR	95% CI
Constipation || id: finn-b-K11_CONSTIPATION	genus.Anaerotruncus.id.2054	88	Inverse variance weighted	0.07	0.03	0.007	1.08	1.02–1.13
			MR Egger	0.17	0.14	0.237	1.18	0.90–1.56
			Weighted median	0.12	0.04	0.001	1.13	1.05–1.21
			Simple mode	0.12	0.09	0.165	1.13	0.95–1.34
			Weighted mode	0.12	0.09	0.181	1.13	0.95–1.35
Constipation || id: finn-b-K11_CONSTIPATION	genus.Butyricimonas.id.945	50	Inverse variance weighted	0.07	0.03	0.015	1.07	1.01–1.13
			MR Egger	0.21	0.12	0.087	1.23	0.97–1.55
			Weighted median	0.08	0.04	0.038	1.08	1.00-1.17
			Simple mode	0.08	0.09	0.381	1.08	0.91–1.28
			Weighted mode	0.08	0.08	0.333	1.08	0.93–1.26
Constipation || id: finn-b-K11_CONSTIPATION	genus.Hungatella.id.11,306	53	Inverse variance weighted	0.03	0.01	0.037	1.03	1.00-1.06
			MR Egger	0.02	0.19	0.899	1.02	0.71–1.48
			Weighted median	0.03	0.02	0.089	1.03	1.00-1.06
			Simple mode	0.03	0.04	0.429	1.03	0.96–1.10
			Weighted mode	0.03	0.03	0.425	1.03	0.96–1.10

### *Ruminiclostridium* 9 and *Intestinibacter* have protective effects of constipation

Our analysis also revealed that *Ruminiclostridium* 9 and *Intestinibacter* were significantly associated with a lower risk of developing constipation. As shown in Tables 2, 169 and 77 SNPs were regarded as the IVs of *Ruminiclostridium* 9 and *Intestinibacter* respectively.

**Table 2:** Mendelian randomization results of protective factors of constipation

Outcome	Exposure	IVs	Method	β	se	pval	OR	95% CI
Constipation || id: finn-b-K11_CONSTIPATION	genus.Ruminiclostridium9.id.11,357	169	Inverse variance weighted	-0.28	0.02	< 0.001	0.75	0.73–0.78
			MR Egger	-0.30	0.18	0.090	0.74	0.52–1.05
			Weighted median	-0.33	0.02	< 0.001	0.72	0.68–0.75
			Simple mode	-0.35	0.07	< 0.001	0.71	0.62–0.80
			Weighted mode	-0.35	0.07	< 0.001	0.71	0.62–0.80
Constipation || id: finn-b-K11_CONSTIPATION	genus.Intestinibacter.id.11,345	77	Inverse variance weighted	-0.11	0.02	< 0.001	0.89	0.86–0.93
			MR Egger	-0.15	0.06	0.020	0.86	0.77–0.97
			Weighted median	-0.16	0.03	< 0.001	0.85	0.80–0.89
			Simple mode	-0.17	0.05	< 0.001	0.85	0.77–0.94
			Weighted mode	-0.17	0.05	< 0.001	0.85	0.77–0.93

**Fig. 3:**
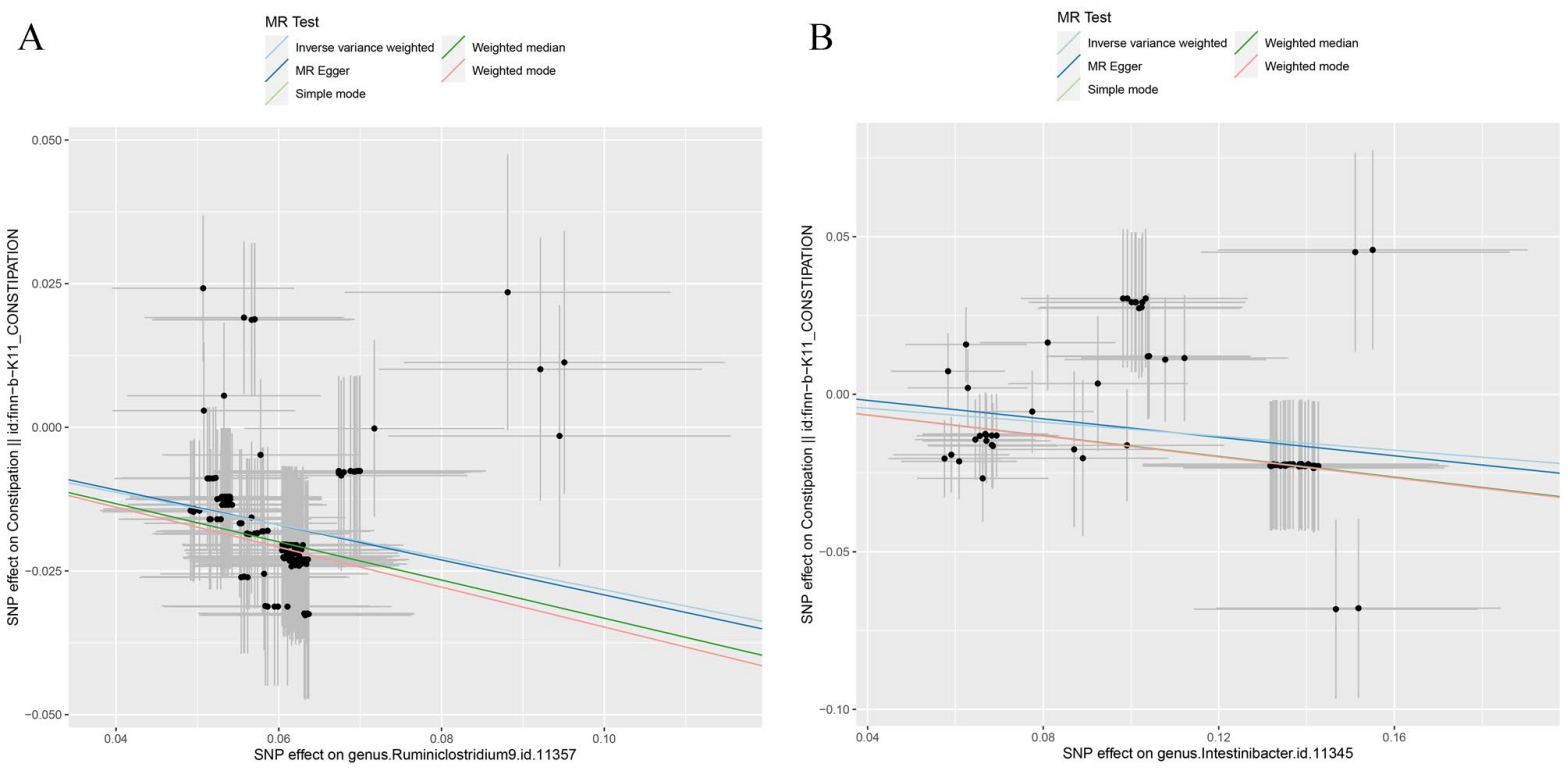
Scatter plots for the causal association between gut microbiota and constipation (**A**) *Ruminiclostridium9* (**B**) *Intestinibacter*

*Ruminiclostridium* 9 showed an odds ratio (OR) of 0.75 (95% CI 0.73–0.78; *P* < 0.001) and *Intestinibacter* an OR of 0.89 (95% CI 0.86–0.93; *P* < 0.001). The other four methods yielded slightly lower ORs and β-values but with wider confidence intervals (95% CI). Scatter plots (Fig. 3A-B, slopes > 0) corroborated these results and demonstrated that no other factors influenced the outcomes, as the intercepts of all five MR methods were close to zero.

### Validation of MR results

**Fig. 4:**
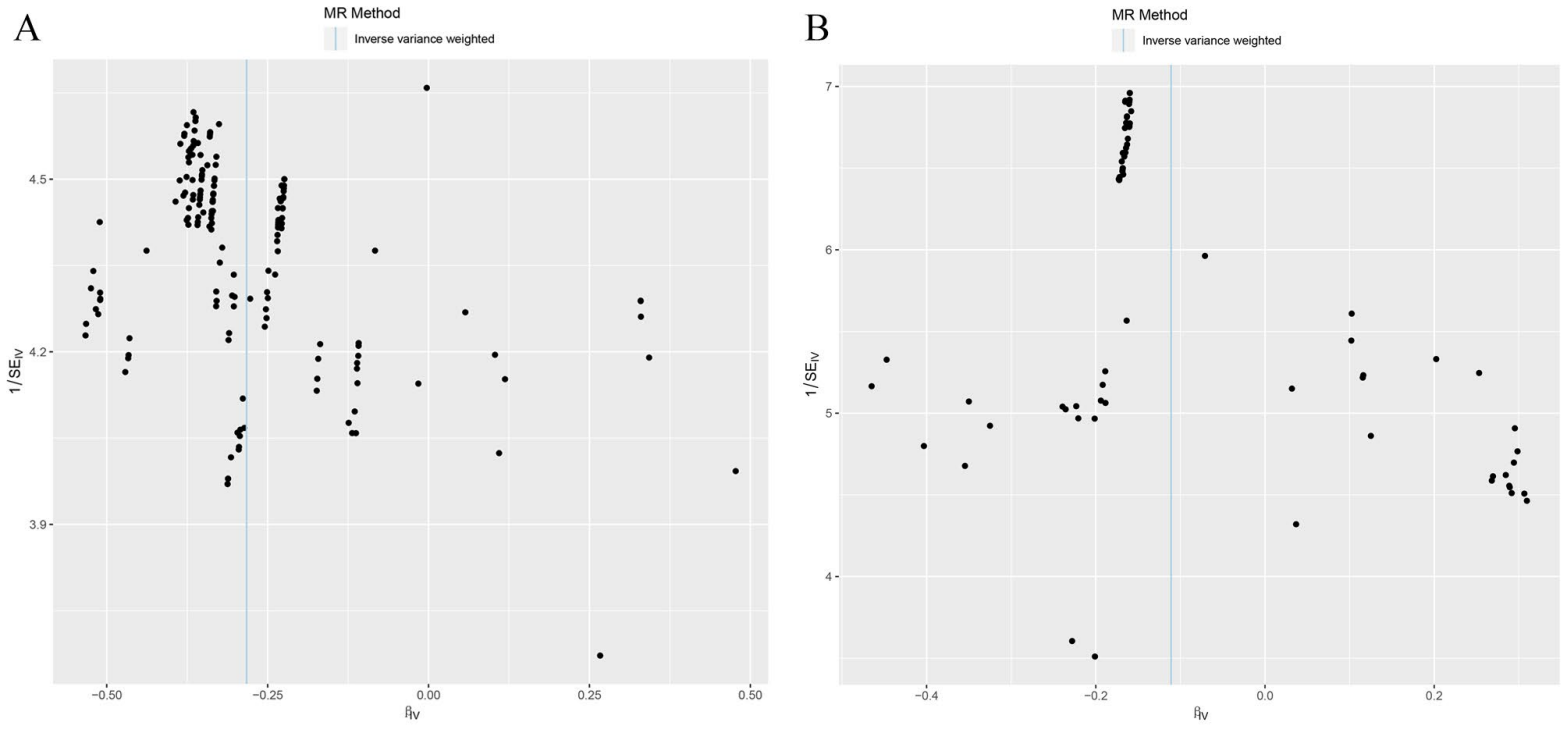
Funnel plot assessing SNP bias in Mendelian randomization analysis

**Fig. 5:**
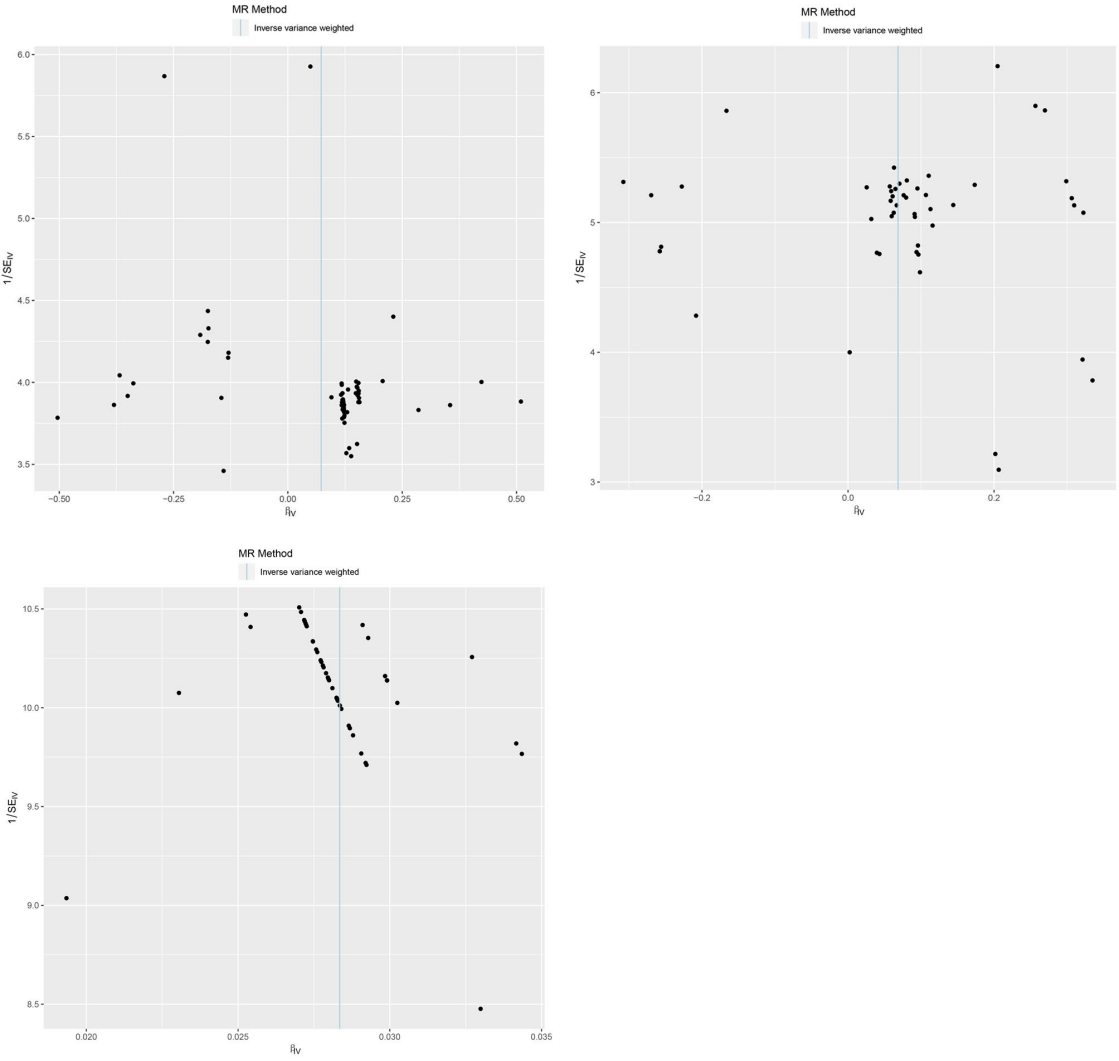
Funnel plot assessing SNP bias in Mendelian randomization analysis

The most IVs of *Ruminiclostridium* 9 were symmetrically distributed in a funnel plot (Fig. 4A), without outlying SNPs (Supplementary Fig. 1A), suggesting the effectiveness of IVs, as well as most IVs of *Intestinibacter* (Fig. 4B, Supplementary Fig. 1B), *Anaerotruncus* (Fig. 5A, Supplementary Fig. 2A), *Butyricimonas* (Fig. 5B, Supplementary Fig. 2B) and *Hungatella* (Fig. 5C, Supplementary Fig. 2C). As shown in Table 3, the heterogeneity test and horizontal pleiotropy test results of *Ruminiclostridium* 9 (Q-pvalue = 0.99; *P* = 0.90), *Intestinibacter* (Q-pvalue = 0.56; *P* = 0.55), *Anaerotruncus* (Q-pvalue = 0.99; *P* = 0.49), *Butyricimonas* (Q-pvalue = 0.96; *P* = 0.23) and *Hungatella* (Q-pvalue = 1.00; *P* = 0.98) suggested that no heterogeneity and horizontal pleiotropy existed. Namely, MR results were reliable.

(A.) *Ruminiclostridium9* (B). *Intestinibacter*.

(A) *Anaerotruncus*, (B) *Butyricimonas and* (C) *Hungatella*.

**Table 3:** Heterogeneity test and horizontal pleiotropy test results

Exposure	Outcome	pval	Method	Q_pval
*Ruminiclostridium*9	Constipation	0.903	Inverse variance weighted	1.000
*Intestinibacter*	Constipation	0.545	Inverse variance weighted	0.560
*Anaerotruncus*	Constipation	0.493	Inverse variance weighted	1.000
*Butyricimonas*	Constipation	0.234	Inverse variance weighted	0.961
*Hungatella*	Constipation	0.981	Inverse variance weighted	1.000

## Discussion

By analyzing gut microbiota summary statistics from the MiBioGen consortium and from the openGAWS consortium on constipation, we carried out a two-sample MR analysis to determine the causal association between gut microbiota and constipation. To our knowledge, current research is the first of its kind to explore the causal association between gut microbiota and constipation. Notably, we found *Ruminiclostridium* 9 and *Intestinibacter* to be potentially protective against constipation, while *Anaerotruncus*, *Butyricimonas*, and *Hungatella* were identified as causally associated with constipation.

Our research established that *Anaerotruncus*, *Butyricimonas*, and *Hungatella* are associated with an increase in the risk for constipation. These bacteria are all anaerobic; *Anaerotruncus* [29] and *Hungatella* [30] are Gram-positive, while *Butyricimonas* [31] are Gram-negative. Consistent with prior studies on non-motor symptoms of Parkinson’s disease (PD), an increased presence of *Anaerotruncus* and *Hungatella* was observed in constipated PD patients [32]. Additionally, *Butyricimonas* showed a significant increase in patients with functional constipation [33]. These observations are in line with our MR analysis, indicating the induction of these gut bacteria can be one of the causes of constipation. However, conflicting evidence also exists. For instance, lower levels of *Anaerotruncus* were found in constipated patients in Japan [34]. To address these inconsistencies, further studies should delve into the metabolic processes of these bacteria and their mechanisms of interaction with human cells. For instance, Liu et al. [15] demonstrated that constipation signifcantly changes the diversity of intestinal microbial communities and affected the fecal metabolites. Mechanistically, there is evidence suggesting a direct link between *Anaerotruncus* and constipation, potentially through interactions with the glycoprotein mucin. Mucin, an essential element of mucus, serves several functions: it lubricates the intestines, aids in stool formation, forms a protective barrier along the intestinal lining, and helps retain water in the bowel. Alterations in mucin content are thought to be a contributing factor in the onset of chronic constipation [35]. Notably, *Anaerotruncus* has been demonstrated to degrade mucin by targeting its carbohydrate chains implying that an increase in *Anaerotruncus* could lead to the breakdown of mucin [36]. Nonetheless, simultaneous investigations of fecal *Anaerotruncus* content and mucin levels in the intestinal wall have not been reported. Paradoxically, *Anaerotruncus* and *Butyricimonas* can produce butyric acid, which is considered to alleviate constipation [37, 38]. Few studies have confirmed that a higher ratio of butyric and acetic acids to total acids is effective in mitigating constipation [39]. Even if constipation patients have an increased presence of Anaerotruncus and Butyricimonas, which can produce butyric acid, it is possible that other butyric acid-producing bacteria may decrease in cases of constipation. Given the presence of numerous gut bacteria capable of producing butyric acid, it is a common byproduct of gut bacterial fermentation, predominantly among Firmicutes [40]. For a more accurate understanding, measuring butyric acid levels in constipation patients and various butyric acid-producing bacteria within the same group is necessary.

Our analysis suggests a notable protective role for *Ruminiclostridium* 9 and *Intestinibacter* against constipation. However, we found no observational studies on the possible negative association between the above two bacterial species and constipation. An indirect connection might be explored by discussing the known metabolic roles of each bacterium. *Ruminiclostridium* is a kind of mesophilic anaerobic cellulolytic bacteria, which derives its energy primarily from the metabolism of plant matter non-cellulosic heteropolysaccharides such as xylan [8, 41]. It has been shown that the levels of *Ruminiclostridium* 9 can change based on dietary composition, with the long-term intake of pork meat proteins increasing *Ruminiclostridium* 9 in the gut content of mice [42]. The abundance of *Ruminiclostridium* 9 was shown to be altered when probiotic or probiotic/prebiotic/essential oil supplement was taken during a subclinical Necrotic Enteritis challenge in broiler chickens [43, 44]. Similarly, the abundance of *Intestinibacter* in the gut has been shown to be closely related to diet composition. Consumption of of health foods such as arabinoxylan and mixed linkage glucans [45] as well as flaxseed oil has been correlated with higher levels *Intestinibacter* [46]. These nutrients are generally believed to help relieve constipation. Beside, the presence of *Intestinibacter* has been found to be beneficial for the production of long-chain fatty acids, coumaric acid, and other metabolites in cruciferous plants [47]. Long-chain fatty acids have been demonstrated to contribute to the enhancement of colonic motility in rats, which in turn might alleviate constipation.

Intriguingly, from a statistical standpoint, the two protective bacteria demonstrate a substantially stronger effect against constipation compared to the three other identified pathogens. According to the IVW method, their protective ORs are approximately 10–20 times higher than those of the pathogenic bacteria, with β values twice as large. This phenomenon is primarily due to the larger number of SNPs selected as IVs for these protective bacteria during MR analysis. This suggests that these beneficial bacteria are associated with more genetic traits. This could be a result of the long-term co-evolution between humans and these bacteria, where humans have evolved specific genetic traits that help maintain these protective bacteria. Such genetic stability might explain why changes in these beneficial bacterial populations are not typically observed in conventional observational studies of constipation. Nevertheless, an imbalance in any of these bacteria, protective or pathogenic, can result in harmful effects [43, 44].

The current study using MR analysis has several strengths: 1) It minimizes the interference of confounding factors in causal inference. Genetic variants of the gut microbiota were sourced from the largest available GWAS meta-analysis, enhancing the robustness of the instruments used in the MR analysis. Both heterogeneity tests and horizontal pleiotropy tests have confirmed the reliability of our results. A two-sample MR design was employed, using non-overlapping exposure and outcome summary-level data to prevent bias. Despite its strengths, this study also possesses a few limitations. Firstly, there is population bias since the GWAS data are derived only from European populations. The diversity of the gut microbiota is closely linked to environmental factors, and its composition varies significantly across different regions, cultures, and countries. For instance, it was found that the gut microbiota of the Japanese population differs markedly from that of other populations [48]. Future research needs to broaden the scope to better understand the relationships between constipation and gut microbiota. Secondly, age and sex are closely associated with both gut microbiota and constipation. However, due to the lack of age-related GWAS data, a mediation analysis was not conducted. It has been reported that *Butyricimonas* may have accelerating effects on Bioage or PhenoAge, which could elucidate the age-related link with constipation [49]. However, the research on this relationship is limited and may require further experimental validation. Thirdly, this study did not perform reverse MR analysis because using constipation as the exposure did not yield significant SNPs for subsequent analysis. This suggests that the genetic markers of constipation are not as robust or clearly defined, indicating lower heritability. Therefore we cannot definitively exclude that the observed increases or decreases in certain bacteria are a result of constipation. Moreover, the analysis was constrained to bacterial taxa at the order or family level. Utilizing more advanced techniques such as shotgun metagenomic sequencing in GWAS could yield more specific and accurate results. Furthermore, other contributing factors to the gut microbiota-constipation link, such as gastrointestinal active peptides, dietary factors, and more among different populations were not taken into consideration from GAWS data.

In summary, our study used MR to find a causal relationship between constipation and differences in specific bacteria within the gut microbiota for the first time. We identified specific microbe families associated with constipation, which affords fresh insights into the pathogenesis of the disease and the possibility of developing treatment strategies. Due to the limitations imposed by the fact that analysis was completed on the order of bacterial family level, we hope that future studies can look deeper into the bacterial families of interest on the level of genus and species to identify the key players concerning constipation. Future studies involving a broader range of racial and ethnic groups are imperative to avoid biased estimates and enhance the universality of our findings. Finally, while MR is an effective method for causal analysis, it’s essential to complement these findings with further basic and clinical experiments to confirm our results and to fully understand the underlying mechanisms.

### Electronic supplementary material

Below is the link to the electronic supplementary material.

Supplementary Material 1

## Data Availability

The data analyzed in this study are sourced exclusively from publicly available datasets. Specifically, we utilized data from the Integrative Epidemiology Unit (IEU) Open GWAS database, accessible at [https://gwas.mrcieu.ac.uk], and the MiBioGen database, available at [www.mibiogen.org].

## Abbreviations

CRC: Colorectal cancer
CV: Cardiovascular disease
ESRD: End-stage renal disease
FPI: Fasting plasma insulin
GWAS: Genome-Wide Association Studies
IEU OpenGWAS: IEU Open Genome-Wide Association Studies
IVs: Instrumental variables
IVW: Inverse-variance weighted
MR: Mendelian randomization
nSNP: Number of single nucleotide polymorphisms
OR: Odds ratio
PPEO: Probiotic/prebiotic/essential oil supplement
PROB: Probiotic
SNPs: Single nucleotide polymorphisms
