# Supplementary material for: The causal relationship between gut microbiota and constipation: a two-sample Mendelian randomization study
Source: BMC Gastroenterol. 2024 Aug 19;24:271. doi: 10.1186/s12876-024-03306-8 (PMC11331768; doi:10.1186/s12876-024-03306-8)
Supplement: Supplementary file 1 — Supplementary Material 1 [file 12876_2024_3306_MOESM1_ESM.pdf]

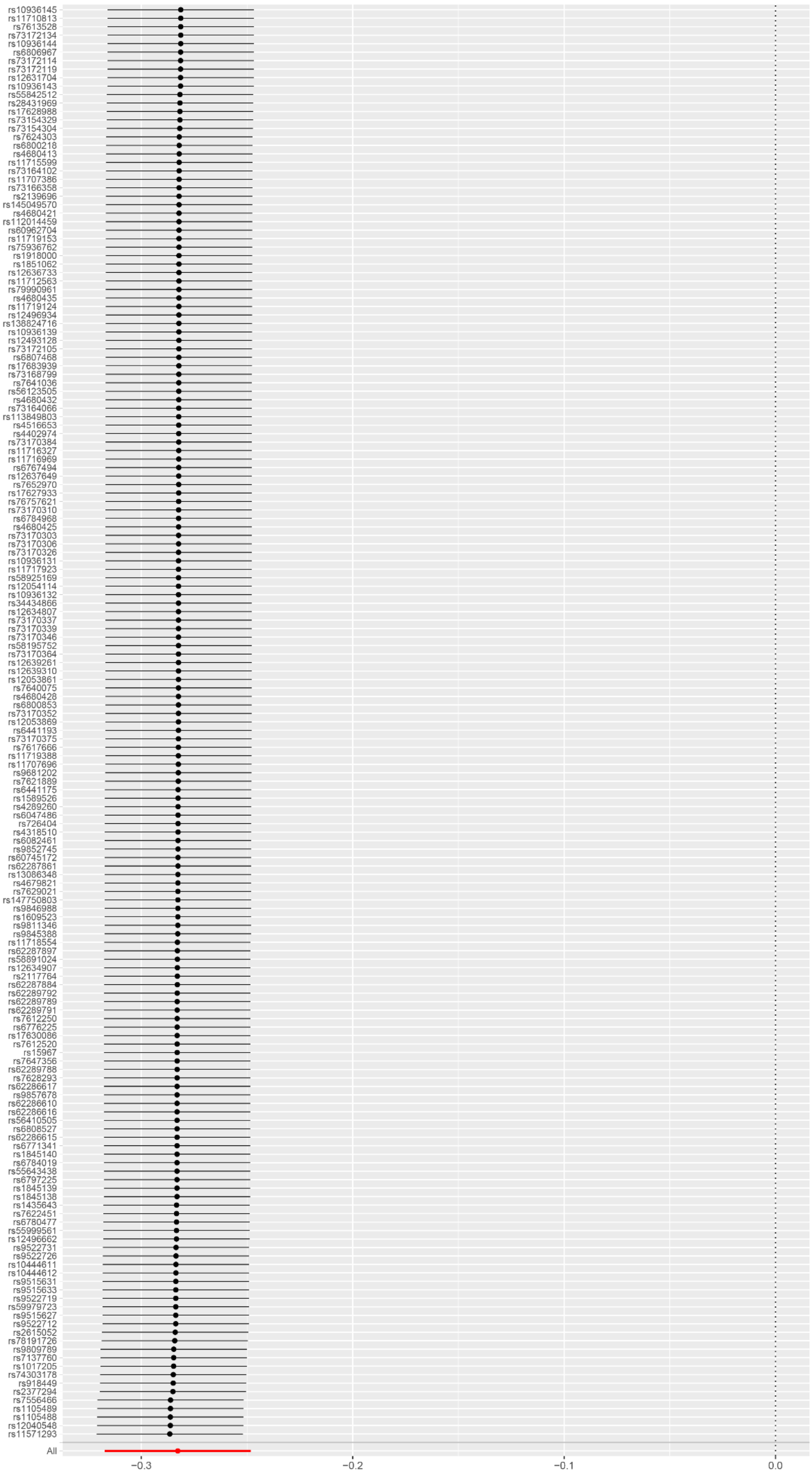

Supplementary Fig.1 MR leave -one-out sensitivity analysis for genus.

Ruminiclostridium9.id.11357' on 'Constipation || id:finn−b−K11_CONSTIPATION

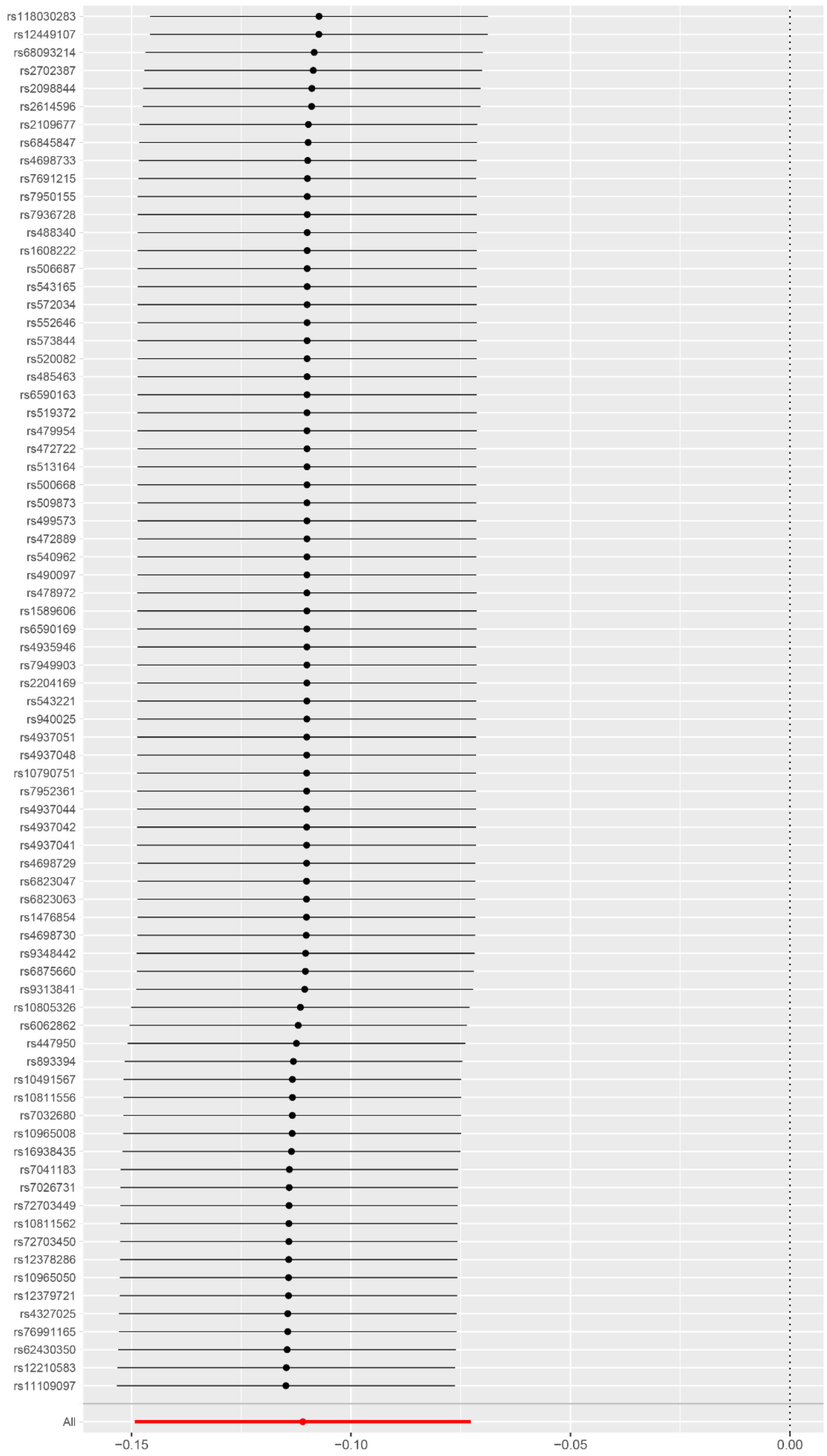

Supplementary Fig.2 MR leave -one-out sensitivity analysis for genus.
Intestinibacter.id.11345' on 'Constipation || id:finn−b−K11_CONSTIPATION

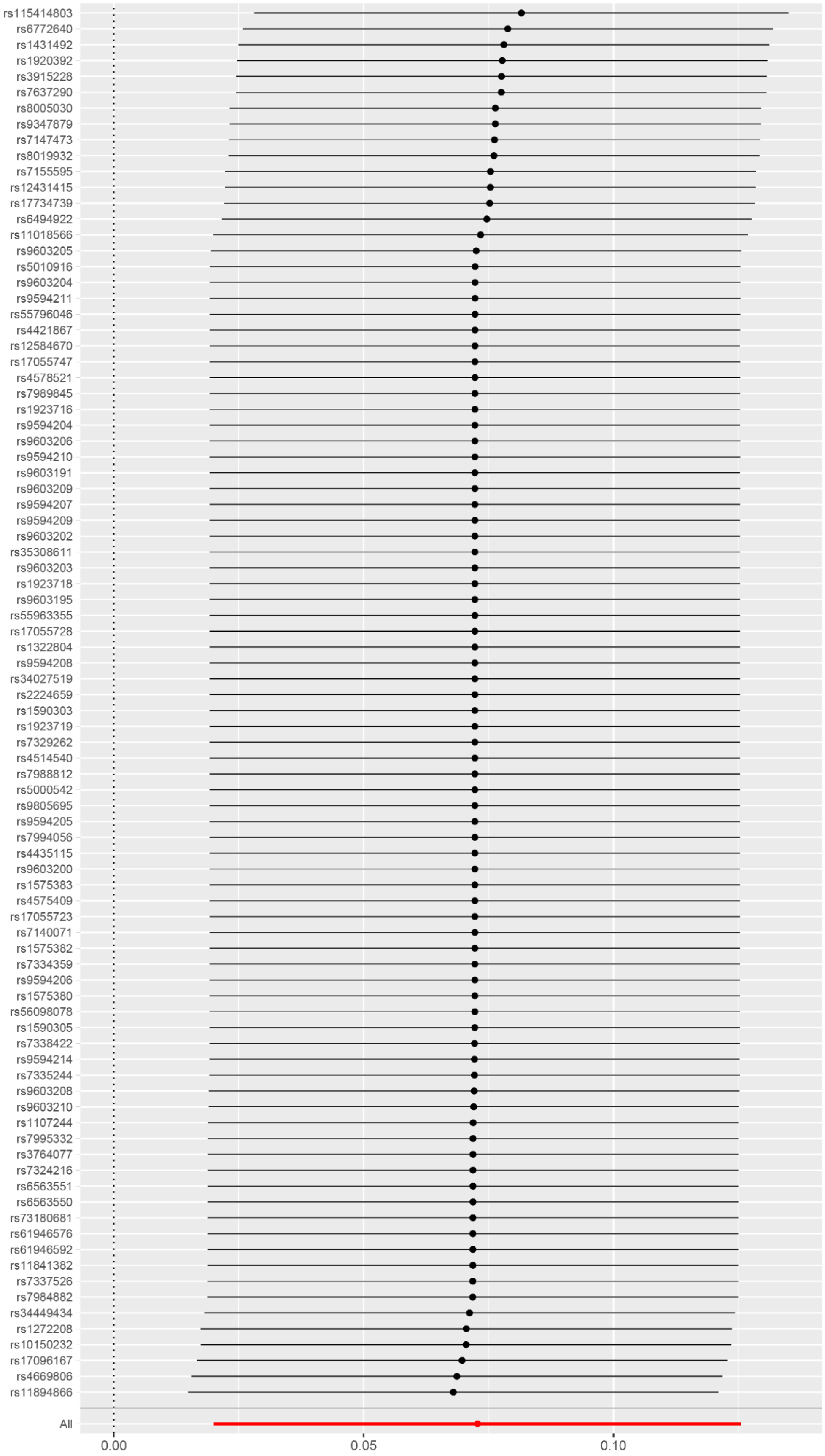

Supplementary Fig.3 MR leave -one-out sensitivity analysis for genus.

Anaerotruncus.id.2054' on 'Constipation || id:finn−b−K11_CONSTIPATION

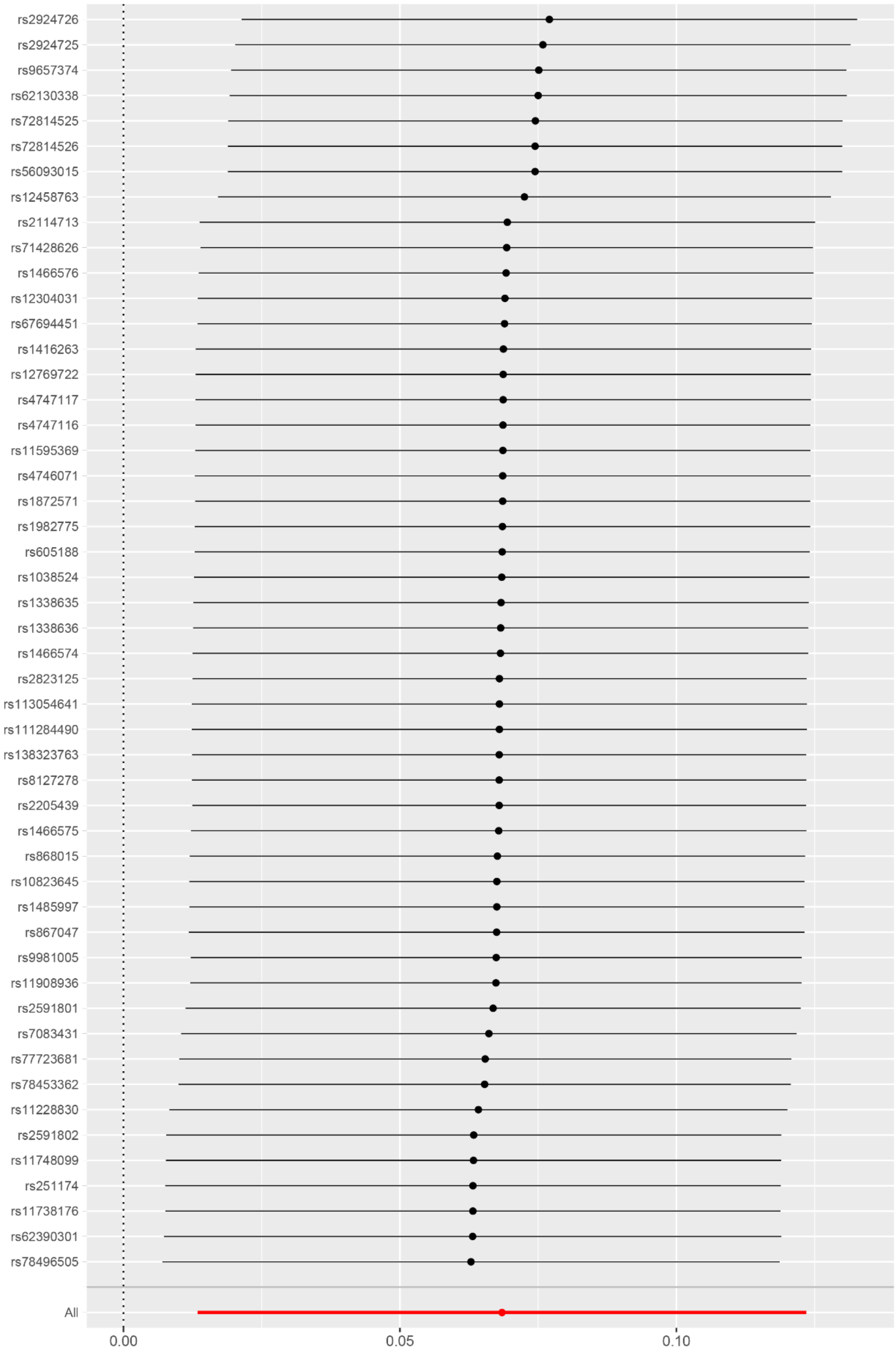

Supplementary Fig.4 MR leave -one-out sensitivity analysis for genus. Butyricimonas.id.945' on 'Constipation || id:finn−b−K11_CONSTIPATION

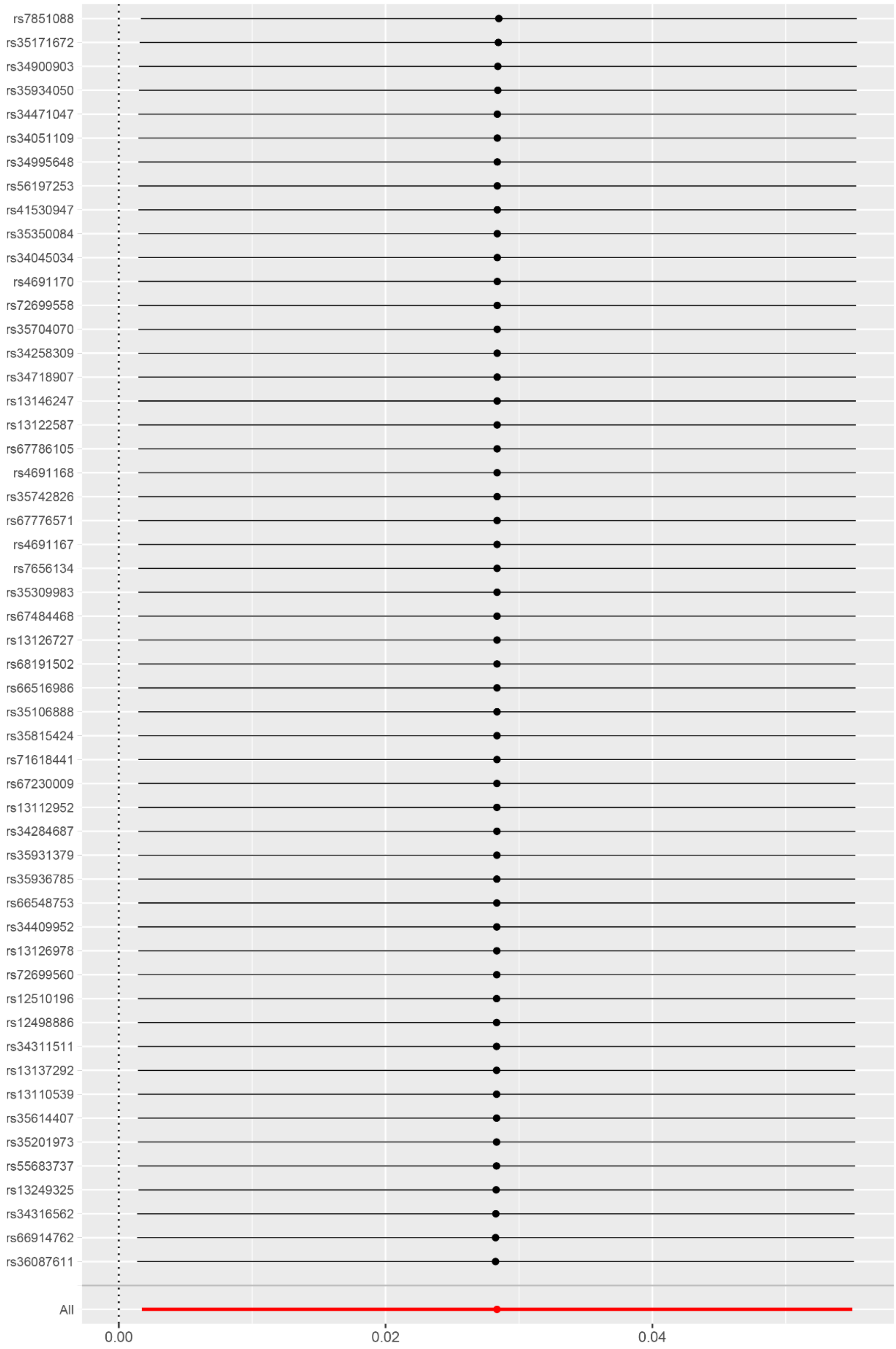

Supplementary Fig.5 MR leave -one-out sensitivity analysis for genus.

Hungatella.id.11306 ' on 'Constipation || id:finn−b−K11_CONSTIPATION
